# The Association of Vitamin D Status with Disease Activity in a Cohort of Crohn’s Disease Patients in Canada

**DOI:** 10.3390/nu9101112

**Published:** 2017-10-12

**Authors:** Dania Alrefai, Jennifer Jones, Wael El-Matary, Susan J. Whiting, Abdulrahman Aljebreen, Naghmeh Mirhosseini, Hassan Vatanparast

**Affiliations:** 1 College of Pharmacy and Nutrition, University of Saskatchewan, Saskatoon, SK S7N 5C9, Canada; dania.alrefai@gmail.com (D.A.); susan.whiting@usask.ca (S.J.W.); 2 Division of Digestive Care & Endoscopy, Department of Community Health and Epidemiology, Dalhousie University, Truro, NS B2N 5E3, Canada; jennifer.jones@nshealth.ca; 3 College of Medicine, University of Manitoba, Winnipeg, MB R3T 2N2, Canada; welmatary@exchange.hsc.mb.ca; 4 College of Medicine, King Saud University, Riyadh 11451, Saudi Arabia; amaljebreen@gmail.com; 5 Pure North S’Energy Foundation, Calgary, AB T2R 0C5, Canada; Naghmeh.Mirhosseini@purenorth.ca

**Keywords:** Crohn’s disease, vitamin D, disease activity, inflammatory bowel diseases

## Abstract

We determined the association between vitamin D status as 25hydroxyvitamin D [25(OH)D] and disease activity in a cohort of 201 Crohn’s Disease (CD) patients in Saskatoon, Canada over three years. The association between high-sensitivity C-reactive protein (hs-CRP) and 25(OH)D and several disease predictors were evaluated by the generalized estimating equation (GEE) over three time-point measurements. A GEE binary logistic regression test was used to evaluate the association between vitamin D status and the Harvey-Bradshaw Index (HBI). The deficient vitamin D group (≤29 nmol/L) had significantly higher mean hs-CRP levels compared with the three other categories of vitamin D status (*p* < 0.05). CRP was significantly lower in all of the other groups compared with the vitamin D-deficient group, which had Coef. = 12.8 units lower (95% CI −19.8, −5.8), Coef. 7.85 units (95% CI −14.9, −0.7), Coef. 9.87 units (95% CI −17.6, −2.0) for the vitamin D insufficient, adequate, and optimal groups, respectively. The vitamin D status was associated with the HBI active disease category. However, the difference in the odds ratio compared with the reference category of deficient vitamin D category was only significant in the insufficient category (odds ratio = 3.45, *p* = 0.03, 95% CI 1.0, 10.8). Vitamin D status was inversely associated with indicators of disease activity in Crohn’s disease, particularly with the objective measures of inflammation.

## 1. Introduction

The prevalence of autoimmune diseases is rapidly increasing around the globe [1]. Inflammatory bowel diseases (IBDs), including ulcerative colitis (UC) and Crohn’s disease (CD), are among the fastest growing autoimmune diseases in developing and developed countries [1]. Canada has one of the highest rates of IBD in the world, due to genetic and environmental factors, which affects 670/100,000 of the Canadian population, in comparison with 27–48/100,000 of the European population [2,3]. Inflammatory bowel disease is characterized by chronic relapsing intestinal inflammation. Dysfunction of the immune system contributes to the improper intestinal inflammatory response in patients with IBD [4].

Crohn’s disease is a disease with a broad spectrum of clinical manifestations, including abdominal pain, severe diarrhea, fatigue, weight loss and malnutrition. It is more common in women, and age at diagnosis is almost 30 [5]. The diagnosis of Crohn’s disease is often challenging. It is primarily based on medical history and physical examination, and supported by a combination of laboratory (C-reactive protein and Fecal calprotectin), biopsy, and endoscopic examination [6,7,8]. The highest incidence of CD is seen in the United Kingdom (UK), North America, and the northern part of Europe [6,7]. The incidence has been reported as 9.2 per 100,000 Finnish [8], 133 per 100,000 in Minnesota, in the United States (US) [9], and 26–198.5 per 100,000 North Americans [10]. In 2012, approximately 233,000 patients had IBD (129,000 with CD and 104,000 with UC) in Canada [4,5]. Left untreated, CD might have serious complications, including bowel obstruction, ulcers, fistulas, and colon cancer. Anti-inflammatory drugs (corticosteroids) are the first step in the treatment of CD [11].

The role of nutrition in preventing disease activity in IBD, and the impact of IBD on the nutritional status of patients, have both been documented in the recent literature [12,13,14]. The absorption of vitamin D in IBD patients is reduced due to the inflammation, disease activity, surgery, or location of the disease, so a high percentage of patients with CD suffer from vitamin D deficiency. It has also been suggested that vitamin D deficiency may have a role in the development of IBD and the disease severity [15,16]. In addition to its several health benefits, vitamin D has an impact on the modulation of the autoimmune system through its effect on the development and function of T cells [12]. The immune system cells can make the active form of vitamin D (1,25(OH)_2_D_3_), which binds to Vitamin D Receptor-Retinoic Acid X Receptor (VDR-RXR) to function in the immune system by decreasing the production of maturation in IL-12, IL-6, and IL-23 (13). On the other hand, the vitamin D receptor is expressed on all immune cells, and its connection with vitamin D leads to the activation of immune cells such as monocytes, macrophages, and neutrophils, resulting in enhanced anti-inflammatory functions, so vitamin D deficiency might be a contributing factor for chronic inflammation [17,18]. Vitamin D deficiency and insufficiency [serum 25(OH)D < 30 and 50 nmol/L, respectively] have been shown to be more common in people suffering from chronic diseases, such as those diagnosed with IBD [19,20]. One of the primary nutritional health issues faced by patients with CD is low vitamin D status [25(OH)D < 50 nmol/L], which can lead to more serious health complications, such as increasing disease activity [21,22,23]. There is some evidence to suggest that vitamin D supplements may be beneficial in treating CD, because higher levels of serum 25(OH)D can significantly decrease disease activity through attenuating inflammatory processes [23,24,25,26]. With the focus on the main biological mechanism involved, which is inflammation, we aimed to determine the association between vitamin D status and disease activity in a cohort of patients with CD, using high-sensitivity C-reactive protein (hs-CRP) as the measure of inflammation. This was a retrospective analysis looking at the 15–40-year old Canadians living in the central part of Canada (Saskatoon, Saskatchewan) and admitting to Royal University Hospital for their course of treatments.

## 2. Methods

### 2.1. Design, Study Site, Data Source, and Ethics Approval

This study was a retrospective cohort study of 201 patients with Crohn’s disease. We extracted the data from medical charts at the IBD clinic of the Royal University Hospital in Saskatoon, Saskatchewan, Canada over three years from 2011 to 2014. This is the only IBD clinic that refers to the university in Saskatchewan and has available data for CD patients. The biomedical research ethics board (Bio 14-161) at the University of Saskatchewan and the Saskatoon Health Region approved the study protocol.

We requested a username and password to access the medical charts at the Royal University Hospital. We collected the data from July to August 2014, and entered them directly into a password-protected Excel spreadsheet. We then stored the data collection file in a university network JADE (JADE Allows Data Exchange) file for Dr. Vatanparast. The data items include age, sex, disease duration, current medications and supplements, history of surgery, Harvey-Bradshaw Index (HBI) scores, and blood test results (e.g., hs-CRP and 25hydroxyvitamin D (25(OH)D)). The laboratory measures are done at the University of Saskatchewan Royal University Hospital biomedical laboratory. For measuring hs-CRP, the Roche CRP assay based on the principal of particle-enhanced immunoturbidimetric assay was used. For the 25(OH)D measurement, Roche Elecys (electrochemiluminescence immunoassay), which employs a vitamin D-binging protein as the capture protein to bind 25(OH)D onto the competition principle, was used. The season of measurements was also taken into account. All of the available data were extracted and entered into the excel spreadsheet, and then divided into three different points in time (first visit, midpoint visit, last visit).

The follow-up visits that were assigned in this study were midpoint and last visits. Midpoint visit occurred eight months after the first visit, and the last visit was the latest visit in patient’s file. The number of months from the first visit was 8.2 ± 5.5 month for the second visit, and 15.3 ± 6.6 month for the third visit. There are two main goals in CD management. The short-term goal is controlling symptoms and starting remission through suppressing inflammation, which was assessed in the midpoint visit. The long-term goal is maintaining remission, which was investigated in the last visit [Crohn’s and Colitis Foundation of Canada (CCFC 2008)].

### 2.2. Measurements

We reviewed medical charts at the Royal University Hospital of the University of Saskatchewan in Saskatoon, Canada. The variables collected included age, gender, disease duration, current medications and supplements, history of surgery, Harvey-Bradshaw Index (HBI) scores, Crohn’s disease activity indicator (CDAI), serum hs-CRP and serum 25(OH)D concentrations.

Anthropometric measurements included height, weight, body mass index (BMI), and waist circumference at baseline. We evaluated the patients’ general physical activities by using the Canadian Health Measures Survey Cycle 2 Physical Activity Questionnaire, 2011.

Patients with Crohn’s disease who scored less than 5 on the HBI were very likely to be in remission, according to the CDAI. The Crohn’s disease activity index is the most widely used tool to measure disease severity and activity in adults, though its validation has been controversial [27,28]. This score does not incorporate a subjective assessment of disease, and does not correlate well with endoscopic factors and systemic features [29]. So, in this study, HBI score was used as the disease indicator. Patients with a score of 5 or higher were considered to have active disease. The severity of disease was defined as mild if the HBI score was greater than 5 and less than 7, moderate with a HBI score between 8 and 16 and severe with a HBI score greater than 16 [30]. The season of measurements was also taken into account, with April to September defined as summer, and October to March as winter. Seasonal changes significantly affect cholecalciferol production. Vitamin D deficiency is more common during the winter period, because the Ultra-violate B (UVB) radiation is not strong enough to produce adequate vitamin D (22–24). Data for each CD case were organized into three time-points based on prior visits: retrospective [first visit (first day of measurement)], midpoint visit = eight months (mean) from the first visit, and final visit (the last measurement available on the data file). We described specific details about the variables used in Table 1.

#### Vitamin D Measurements

Vitamin D supplement intake was reported in 48 patients, with a mean of 1629 ± 1190 IU/day, as most of them were taking a daily vitamin D supplement of 1000 IU as a minimum dose. The official daily recommended amount of vitamin D has been 600–800 IU/day; however, due to a high incidence of vitamin D deficiency in North America, 1000–2000 IU/day is routinely used (Health Canada 2010). At the first visit, 116 patients had their serum vitamin D measured between April and September (summer), and 86 patients had their measurements between October and March (winter). At the midpoint, 107 patients had their measurements between October and March. Most of the patients (*n* = 114) had their last visit between April and September.

### 2.3. Statistical Analysis

We used descriptive statistics and summarized the data as means and standard deviation (SD) for continuous variables and percentages for categorical variables at three time-points, including first visit, midpoint, and endpoint, using SPSS Version 22 (IBM, New York, NY, USA, 2014). We conducted a non-parametric test (Kruskal-Wallis) post hoc to test the potential differences between the variables of interest across the three time-points.

In this current study, the exchangeable correlation matrix between hs-CRP (presented as a continuous variable), the main outcome, and several predictors were evaluated by generalized estimating equation (GEE) at three time points of measurements. In the second model, *HBI* disease score was the main outcome. *HBI* <5 was considered as remission (0), and a score ≥5 was considered as active disease (1). The predictors for *HBI* score and hs-CRP were age, sex, three of the measurements, vitamin D level, and disease duration, use of corticosteroid, surgery, and season. So, all of the models were controlled for potential covariates. Analytical analyses were conducted by STATA/SE 11; StataCorp and alpha was set at 0.05. GEE is a convenient approach to estimate the possible unknown correlation and changes between outcomes over time that have an impact on covariates (25). We used GEE exchangeable correlation matrix with an hs-CRP variable using the following model: (xtgee hs-crp age sex disease_duration i. times, surgery i. vitd_cat4 cs season, family (gaussian) link (identity) corr (exchangeable) eform). To evaluate the association between vitamin D and HBI in the presence of other covariates, GEE with logistic regression was used, with a cut-off HBI score of ≥5, as a categorical variable using the following model: (xtgee hbi_2cat age sex disease_duration i. times surgary i. vitd_cat4 cs season, family (binomial 1) link (logit) corr (exchangeable) eform).

## 3. Results

### 3.1. Descriptive Analysis

#### 3.1.1. Socioeconomic, Demographic, Disease Duration, Surgery, and Corticosteroids

Table 2 shows the general characteristics of the patients. Overall, 42.3% of the patients were male. The mean age was 40.2 ± 15.2 years at baseline, and 41.5 ± 15.1 years at the endpoint. The mean duration between the first (baseline) and second (midpoint) visits was 8.2 ± 5.5 months, and between the second visit and the final visit was 15.3 ± 6.6 months. The mean of the CD duration at the first visit was 9.7 ± 9.6 years. We found that 35.8%, 37.3%, and 39.4% of patients had CD-related surgery at each of the three time-points, respectively. Only 19.4% of patients were placed on corticosteroids in the first visit, and 14.3% in the last visit.

#### 3.1.2. Vitamin D Status

The mean serum vitamin D level was significantly different between the baseline and final visit (*p* = 0.04). At the baseline measurement, the mean serum 25-hydroxyvitamin D [25(OH)D] was 58.2 ± 30.0 nmol/L: 26% of the patients had optimal levels, while 26% had insufficient levels, and 18% of the patients had vitamin D deficiency.

At the midpoint measurement (time 2), the mean serum 25(OH)D was 60.1 ± 31.2 nmol/L: 31.3% of the patients had optimal levels, 31.3% had insufficient levels, and 15.2% of patients were vitamin D deficient.

During the last visit, 66 patients had vitamin D results with the mean of 74.5 ± 42.6 nmol/L: 44% of patients had optimal levels of vitamin D, though 18.1% of patients had insufficient vitamin D levels, and 13.6% had vitamin D deficiency.

At the final visit, 47 patients (28.0%) reported taking a vitamin D supplement, with the mean intake of 2085 ± 1501 IU/day. It was significantly higher from the average of vitamin D intake at the baseline visit (1629 ± 1190 IU/day).

#### 3.1.3. Disease Biomarkers (CRP) and HBI Scores

The mean hs-CRP concentration varied significantly between the baseline and final visit (*p* = 0.001). At baseline, 192 patients had serum hs-CRP recorded, with a mean of 13.0 ± 22.3 mg/L, and 55.7% of them had high-risk levels of hs-CRP (>3 mg/L). Among patients who had reported HBI scores (*n* = 187), 3.2% had a severe disease, 31% were mild cases, 33.1% had a moderate disease, and 32.1% were in remission. At the midpoint, 48.2% of patients had very high levels of hs-CRP, 30.2% had a moderate level, and 21.5% had low levels of hs-CRP. The mean of the hs-CRP levels decreased significantly over time (*p* = 0.001) from the baseline to the final visit, 13.0 ± 22.3 mg/L to 7.2 ± 15.4 mg/L, respectively. Of the patients tested, 38.6% had high levels of hs-CRP at the final visit. Close to half of the patients (*n* = 35 from 67 patients) were in remission, based on their HBI score (Table 3).

### 3.2. Statistical Modeling

#### 3.2.1. High-Sensitivity C-Reactive Protein (hs-CRP)

Table 4 shows the estimates of the model parameters, with 95% confidence intervals (CI) along with results of the tests for their statistical significance. Out of eight covariates included in the model, only three predictors showed a statistically significant association with hs-CRP level. These predictors were corticosteroids, time of measurement, and vitamin D categories. Age, sex, disease duration, season, and surgery did not show a statistically significant effect (*p* > 0.05) on the hs-CRP level.

The estimated coefficient for corticosteroids use was 7.2, with 95% CI of (1.0, 13.5). Time of visit also showed a significant association with hs-CRP. In the final visit, patients had a significantly lower mean hs-CRP level compared with the baseline visit (Coef. = −9.2, *p* = 0.013). However, no significant difference in hs-CRP level was found between the baseline visit and the midpoint visit, which was measured approximately eight months after the baseline. Vitamin D status showed a significant association with hs-CRP level. Vitamin D status was included in the model as a categorical variable: deficient (reference) insufficient, adequate, and optimal levels. All three categories showed significantly lower mean hs-CRP levels compared with the deficient vitamin D category (*p* < 0.05). The insufficient vitamin D category had a Coef. = 12.8 units lower, while the adequate vitamin category had a Coef. = 7.8 units, and the optimal vitamin D category had a Coef. = 9.8 units of the hs-CRP level, which was lower than the deficient vitamin D category. In summary, vitamin D deficiency was significantly associated with a higher hs-CRP level than all of the other three categories of vitamin D status.

#### 3.2.2. Harvey-Bradshaw Index Indicator Category (HBI)

A GEE binary logistic regression test was used to explore the possible association between selected predictor variables and the likelihood of active disease (cut-off ≥ five scores). Table 5 shows the estimates of the odds ratio for each predictor, along with results of the test for their significance. Three predictor variables: (i) use of corticosteroids; (ii) time of visit; and (iii) vitamin D status, had a significant association with the odds of active disease. The use of corticosteroids had an odds ratio estimate of 5.5 and 95% CI (2.1, 14.3). The midpoint visit had an odds ratio of 0.42, *p* = 0.025, 95% CI (0.2, 0.8). This means that the odds of having an active disease during the midpoint visit were 58% lower than odds at the baseline visit. The final visit had an odds ratio of 0.29, *p* = 0.014. This means that odds of having an active disease during the final visit were 71% lower than the odds at the baseline visit.

Vitamin D status was also associated with disease activity. However, only the insufficient category was significantly associated with HBI. The insufficient category had an odds ratio estimate of 3.4 and a 95% CI (1.0, 10.8). For those with vitamin D insufficiency, the odds of having an active disease was 3.4 times higher than the reference group (vitamin D deficient), while the vitamin D deficient participants had elevated hs-CRP. Adequate and optimal vitamin D status did not show any significant difference in the odds of having an active disease compared with deficient vitamin D status.

## 4. Discussion

A large number of CD patients are vitamin D deficient, and suffer from degrees of inflammation. Inflammation keeps Crohn’s disease active. The remission phase is initiated using CD medication. Vitamin D has prominent anti-inflammatory and immunomodulatory properties that make it a favorable case for attenuating inflammation in CD patients. A negative association between improving vitamin D status with inflammation and CD activity was also there.

### 4.1. Vitamin D Status, Supplementation

We found high rates of vitamin D deficiency/insufficiency among active CD patients, with an increased prevalence of vitamin D deficiency during the winter season. Forty-four percent of CD patients had serum vitamin D levels below 50 nmol/L. Our results are concordant with other studies, which indicate that vitamin D deficiency is more common among IBD patients than it is in the general population [31,32,33]. The incidence of vitamin D deficiency and insufficiency (plasma vitamin D below 50 and 75 nmol/L, respectively) have been as high as 62% and 88% among IBD patients (56 CD and 45 UC), respectively [34,35]. The activity of Crohn’s disease is negatively associated with serum vitamin D levels. Comparing active CD patients with those in clinical remission, active disease patients had lower serum vitamin D concentrations [16,36,37].

We found a higher incidence of vitamin D deficiency in the winter season. Our results were consistent with other studies, which show that vitamin D deficiency/insufficiency is more common in the winter season (57%) rather than in the summer season (39%) in IBD patients [38]. Jorgensen et al. (2013) [36] found that in the winter season, CD patients reported ingesting vitamin D supplements (400–800 IU/day) and had significantly higher levels of 25(OH)D (77 nmol/L) than non-users (44 nmol/L). In the summer season, no statistically significant difference was observed between supplement users (80 nmol/L) and non-users (86 nmol/L). So, this indicated that vitamin D supplementation may raise 25(OH)D levels to summer season levels during the winter season in CD patients.

An inverse relationship was found between serum vitamin D levels and inflammatory biomarkers (34). Vitamin D tends to reduce markers of inflammation, which lead to better CD outcomes. Serum vitamin D is significantly associated with hs-CRP levels. The patients with CD who had an optimal vitamin D levels had lower hs-CRP levels (lower disease activity) compared with the vitamin D deficient group.

Vitamin D_3_ supplementation has shown to be safe and well-tolerated in patients with CD [26]. Daily vitamin D_3_ supplementation up to 400 IU did not significantly increase vitamin D concentration in patients with CD. Vitamin D_3_ supplementation up to 2000 IU/day for six months elevated plasma 25(OH)D levels above 30 ng/mL (75 nmol/L) more efficiently than 400 IU/day at six months [39]. A study by Pappa et al. found that a daily vitamin D_2_ supplementation of up to 2000 IU for six weeks was insufficient to maintain the optimal levels in children and adolescents with IBD, while the combination of 2000 IU/day of vitamin D_3_ and 50,000 IU/week increased plasma vitamin D concentration to the optimum level [40].

Boothe et al. (2011) found the doses of 10,000 IU/day had significant results in improving serum vitamin D status when comparing 25(OH)D levels at eight weeks (54.4 ± 22.8 nmol/L) and after 26 weeks of treatment (64.2 ± 29.8 nmol/L) than 1000 IU/day [41]. In our cohort, vitamin D levels improved significantly (*p* = 0.005) from baseline (58 ± 30 nmol/L) to the final visit 15 months later (74.5 ± 42.6 nmol/L), with the disease activity and hs-CRP level both decreasing significantly. A significant increase in vitamin D concentrations over the 15 months was found in our CD cases, who were receiving vitamin D supplements with a mean intake of 1629 IU/day at baseline and 2085 IU/day at the final visit. However, vitamin D supplementation dose and serum 25(OH)D level were not adequate enough, and 32% of participants were still vitamin D insufficient or deficient at the last visit. At the same time, just half of the patients (52%) were in remission, and the other half were still was suffering from some degree of disease. This reinforces the point in the Pappa and Boothe studies that higher doses might have better results.

### 4.2. C-Reactive Protein and Disease Activity Index

High sensitivity C-reactive protein was significantly associated with vitamin D levels among CD patients (*p* < 0.05). Active Crohn’s patients, based on endoscopy results, had elevated levels of hs-CRP and below the optimal concentrations of plasma vitamin D (<75 nmol/L). The disease activity indicator (hs-CRP) decreased significantly over 15 months (*p* = 0.001) in our cohort, while vitamin D concentration increased significantly (*p* = 0.04). The hs-CRP concentrations perhaps increased in some cases because of other factors such as using corticosteroids, which showed a positive association with hs-CRP levels in our cohort (*p* = 0.023, Coef. 7.2). Steroids were prescribed for the short term to reduce the inflammation. However, long-term corticosteroid use results in decreasing calcium absorption and vitamin D metabolism, which contribute to developing osteoporosis [42,43]. In addition, all of the vitamin D categories (deficient category as the reference category) have shown a negative association with hs-CRP levels. A study found oral vitamin D_3_ supplementation up to 1200 IU/day for three months significantly increased vitamin D levels from a mean of 69 to 96 nmol/L, and insignificantly decreased the disease activity from 29% to 13% (*p* = 0.06) [26]. Researchers showed inactive CD patients who were treated with 0.25 μg alfacalcidol (1,25(OH)2D3) for six weeks had a significant reduction in disease activity and plasma CRP concentrations from an average of 15.8 ± 23.57 mmol/L to 7.81 ± 3.91 mmol/L [44].

The Harvey-Bradshaw Index was the only indicator to estimate subjectively the disease activity in our study. In our cohort, the HBI scores showed a significant association with insufficient vitamin D levels. Clinically, we found that 18.2% of CD cases had an active disease at baseline compared with 33% were in remission, according to the HBI score. The HBI had an inverse relationship with clinical biomarkers such as hs-CRP; however, other studies showed that the CDAI might not be correlated with ileocolonic inflammation; Crohn’s patients who were in remission had active disease (colonic inflammation) by endoscopy, and vice versa [45]. Only a small portion of the patients (38 patients out of 131) were in both clinical and endoscopic remission [45]. The heavily weighted symptoms in CDAI and HBI such as general well-being, abdominal pain, and diarrhea were not considered as specific biomarkers of intestinal inflammation [45]. In concordance with previous studies, we found the results of hs-CRP to be the outcome variable, which was more interpretable than the subjective tools. This indicates the importance of using a more objective measure of disease activity in CD patients.

Disease duration and surgery did not reach statistical significance with hs-CRP levels and HBI scores. Our results were consistent with other studies that indicated that CRP levels during a relapse were not significantly associated with surgeries and the duration of disease [46]. However, we found that HBI score was positively associated with the use of corticosteroids (*p* < 0.001, OR = 5.5) and an insufficient vitamin D category (*p* = 0.034, OR = 3.4) in our cohort. Using oral corticosteroids was positively correlated with vitamin D deficiency. Corticosteroid users were more vitamin D deficient (<25 nmol/L) compared with the normal levels group (>50 nmol/L) [47]. In addition, the use of corticosteroid drugs to achieve remission in active disease has been suggested by others [48].

The main limitation of this study was the assay method used to measure serum 25(OH)D levels. Although the gold standard method to measure serum 25(OH)D concentration is Liquid Chromatography-Mass Spectrometry (LC-MS/MS), Leino et al. (2008) [49] showed in their study, which compared different methods of serum 25(OH)D measurements, that electrochemiluminescence immunoassay, which was used in this study, represented a satisfactory overall agreement with LC-MS/MS. There might be a misclassification of participants having serum 25(OH)D levels at the lower end of the measuring range due to a lack of sensitivity [49]. So, there is a chance of misclassifying those who were vitamin D deficient in this study. Another limitation was the retrospective nature of the data, which limited us to only the available information in patients’ charts, with some missing data. Although there was a significant association between vitamin D status and CD activity, the design of the study does not permit the authors to conclude that vitamin D deficiency is detrimental for CD patients. We used the GEE method to overcome the challenge with missing data by considering that they were missing completely at random. Also, we used inflammatory markers such as hs-CRP to correlate with HBI disease activity scores.

In summary, vitamin D deficiency was an outcome of active inflammation in CD patients. Almost half of the CD patients were vitamin D deficient. On the other hand, more than 80% of patients with CD suffered from an intermediate to a high degree of inflammation. Vitamin D concentrations showed a significant inverse association with both hs-CRP level and disease activity levels. Therefore, it seems beneficial to avoid vitamin D deficiency in CD patients.

## 5. Recommendation for Future Research and Practice

The vitamin D status of CD patients should be assessed regularly, and recommendation for vitamin D supplementation should be provided accordingly. The role of vitamin D in autoimmune diseases and the possibility of higher requirements to vitamin D in CD patients warrant further research with higher doses of vitamin D. Additional research/evidence are needed to understand how the pattern of changes in vitamin D status affect the prognosis of CD, and how different doses of vitamin D supplemntation affect the disease activity in CD. Also, large randomized clinical trials are required to understand the impact of vitamin D supplements, especially at higher doses than what is currently recommended to general population, such as 4000 IU and 10,000 IU/day, in CD patients in different ethnicities and environmental factors.

## Figures and Tables

**Table 1:** Definitions of Variables Used in the Data Analysis.

Variable	Type	Description
Age	Continuous	At each time point
Sex	Categorical	Male, female
Disease duration	Continuous	Duration of CD from the day of diagnosis
Times	Categorical	Time 1 = Baseline, Time 2 = Midpoint Time 3 = Endpoint
Months	Continuous	Number of months from the first visit
HBI score	Continuous/Categorical	1. Remission < 5 2. Active disease ≥ 5
History of surgery	Categorical	Yes, No
hs-CRP level	Continuous/Categorical	3. Low-risk < 1 4. Intermediate risk between 1–3 5. High risk > 3
25(OH)D level	Categorical	1. Vit D deficient < 30 nmol/L 2. Vit D insufficient 30–50 nmol/L 3. Vit D adequate 51–74 nmol/L 4. Vit D optimal > 75 nmol/L
Vitamin D supplementation	Continuous	Amount of vitamin D (IU/day)
Season	Categorical	1. April to September (“summer”) 2. October to March (“winter”)
Corticosteroid drugs use	Categorical	Yes, No

HBI: Harvey-Bradshaw index; Hs-CRP: High-sensitivity C-reactive protein; 25(OH)D: 25hydroxyvitamin D.

**Table 2:** Descriptive Characteristics for the Retrospective Cohort Data Demographics, Disease Duration, Surgery and Corticosteroids Variables.

Variables	Time 1	Time 2	Time 3
Age (year) *	(*n = 201*)	(*n = 201*)	(*n = 201*)
	40.2 ± 15.2	40.8 ± 15.2	41.5 ± 15.1
Sex	(*n = 201*)	(*n = 201*)	(*n = 201*)
Male	83 (41.3)	83 (41.3)	83 (41.3)
Female	118 (58.7)	118 (58.7)	118 (58.7)
Number of months from the first visit *	-	8.2 ± 5.5	15.3 ± 6.6
History of surgery	(*n = 201*)	(*n = 201*)	(*n = 180*)
Yes	72 (35.8)	75(37.3)	71 (39.4)
No	129 (64.2)	126 (62.7)	109 (60.6)
Disease duration (year) *	(*n = 162*)	(*n = 162*)	(*n = 139*)
	9.7 ± 9.6	10.5 ± 9.6	11.2 ± 9.8
Corticosteroids	(*n = 196*)	(*n = 196*)	(*n = 175*)
Not using corticosteroids	158 (80.6)	162 (82.6)	150 (85.7)
Using corticosteroids	38 (19.4)	34 (17.3)	25 (14.3)

* Age, disease duration, and number of months from the first visit presented as mean ± SD, the rest presented as number (%).

**Table 3:** Descriptive Characteristics for the Retrospective Cohort Data for Vitamin D Status, Dosage, and Disease Activity Variables.

Variables	Time 1	Time 2	Time 3
Serum 25(OH)D status (nmol/L)	(*n = 116*)	(*n = 118*)	(*n = 66*)
	**58.2 ± 29.9**	60.1 ± 31.2	**74.5 ± 42.6**
Vitamin D categories	(*n = 116*)	(*n = 118*)	(*n = 66*)
Deficient	21 (18)	18 (15.2)	9 (13.6)
Insufficient	30 (26)	37 (31.3)	12 (18.1)
Adequate	35 (30)	26 (22.1)	16 (24.2)
Optimal	30 (26)	37 (31.3)	29 (43.9)
Vitamin D doses (IU/day)	(*n = 48*)	(*n = 56*)	(*n = 47*)
	1629 ± 1190	2095 ± 1860	2085 ± 1501
hs-CRP( mg/L)	(*n = 192*)	(*n = 195*)	(*n = 158*)
	**13.0 ± 22.3**	11.2 ± 28.9	**7.2 ± 15.4**
hs-CRP categories	(*n = 192*)	(*n = 195*)	(*n = 158*)
Low risk	35 (18.2)	42 (21.5)	49 (31)
Intermediate risk	50 (26)	59 (30.2)	48 (30.3)
High risk	107 (55.7)	94 (48.2)	61 (38.6)
HBI score	(*n = 187*)	(*n = 90*)	(*n = 67*)
	7.0 ± 4.4	6.3 ± 4.8	6.0 ± 5.2
HBI score categories	(*n = 187*)	(*n = 90*)	(*n = 67*)
Remission	61 (32.6)	40 (44.4)	35 (52.2)
Mild disease	58 (31)	17 (18.8)	10 (14.9)
Moderate disease	62 (33.1)	29 (32.2)	18 (26.8)
Severe disease	6 (3.2)	4 (4.4)	4 (5.9)
Season	(*n = 201*)	(*n = 201*)	(*n = 168*)
April to September	115 (57.2)	94 (46.8)	114 (67.8)
October to March	86 (42.8)	107 (53.2)	(32.1)

**Table 4:** Factors Associated with Hs-CRP Levels among Crohn’s Disease Patients.

hs-CRP Continuous Variable	Coefficient (Coef.)	95% Confidence Interval	* *p*-Value
Age (year)	−0.1	(−0.3, 0.1)	0.449
Sex	−4.5	(−11.4, 2.3)	0.194
Disease duration (year)	0.2	(−0.1, 0.6)	0.185
Corticosteroids (yes)	**7.2**	**(1.0, 13.5)**	**0.023**
Season (winter)	3.7	(−0.6, 8.1)	0.092
Surgery	0.1	(−7.8, 8.2)	0.968
Times (Time 1 as reference)			
Time 2	−2.7	(−7.2, 1.6)	0.224
Time 3	**−9.2**	**(−15.1, −3.4)**	**0.002**
Vitamin D categories			
Insufficient	**−12.8**	**(−19.8, −5.8)**	**0.000**
Adequate	**−7.8**	**(−14.9, −0.7)**	**0.031**
Optimal	**−9.8**	**(−17.6, −2.0)**	**0.013**

* *p*-value was determined using the generalized estimating equation (GEE) test. *p* < 0.05 was considered to be significant.

**Table 5:** Factors Associated with HBI Scores among Crohn’s Disease Patients.

HBI with Two Categories	Odds Ratio (OR)	95% Confidence Interval	* *p*-Value
Age	0.9	(0.9, 1.0)	0.778
Sex	0.7	(0.3, 1.6)	0.505
Disease duration	1.0	(0.9, 1.0)	0.910
Corticosteroids (yes vs. no)	**5.5**	**(2.1, 14.3)**	**0.000**
Season (winter vs. summer)	0.9	(0.4, 1.9)	0.916
Surgery	0.6	(0.2, 1.6)	0.398
Times (Time 1 as ref.)			
Time 2	**0.4**	**(0.2, 0.8)**	**0.025**
Time 3	**0.2**	**(0.1, 0.7)**	**0.014**
Vitamin D categories (Deficient as ref.)			
Insufficient	**3.4**	**(1.0, 10.8)**	**0.034**
Adequate	1.0	(0.3, 2.9)	0.919
Optimal	2.7	(0.9, 8.2)	0.072

* *p*-value was determined using a GEE logistic regression test. *p* < 0.05 were considered to be significant. HBI scores with two cut-off categories (0 ≤ 5 remission and 1 ≥ 5 active disease).
